# Questioning Racial and Gender Bias in AI-based Recommendations: Do Espoused National Cultural Values Matter?

**DOI:** 10.1007/s10796-021-10156-2

**Published:** 2021-06-20

**Authors:** Manjul Gupta, Carlos M. Parra, Denis Dennehy

**Affiliations:** 1 Florida International University, Miami, FL USA; 2 NUI Galway, Galway, Ireland

**Keywords:** Artificial intelligence, Recommender systems, Culture, Racial bias, Gender bias, Responsible AI, Algorithmic bias, Ethical AI

## Abstract

One realm of AI, recommender systems have attracted significant research attention due to concerns about its devastating effects to society’s most vulnerable and marginalised communities. Both media press and academic literature provide compelling evidence that AI-based recommendations help to perpetuate and exacerbate racial and gender biases. Yet, there is limited knowledge about the extent to which individuals might question AI-based recommendations when perceived as biased. To address this gap in knowledge, we investigate the effects of espoused national cultural values on AI questionability, by examining how individuals might question AI-based recommendations due to perceived racial or gender bias. Data collected from 387 survey respondents in the United States indicate that individuals with espoused national cultural values associated to collectivism, masculinity and uncertainty avoidance are more likely to question biased AI-based recommendations. This study advances understanding of how cultural values affect AI questionability due to perceived bias and it contributes to current academic discourse about the need to hold AI accountable.

## Introduction

There are numerous examples of how artificial intelligence (AI) may impact and enhance traditional business functions such as marketing (Wedel & Kannan, 2016), finance and accounting (Mirzaey, Jamshidi, & Hojatpour, 2017), supply chain (Min, 2010) and inventory management (Sustrova, 2016). All the way to improving human resources, for example, in relation to recruitment (van Esch & Black, 2019), as well as how it may help facilitate innovation (Paschen et al., 2020) and public sector implementations (Desouza et al., 2020). Indeed, many companies are intent on exploiting the potential of AI, not just because doing so may contribute $13 trillion to the global economy in the coming decade (Fountaine et al., 2019), but mainly because adopting AI should no longer be considered an option but a necessity for managers and businesses in general.

Most accounts of the evolution of AI tend to place its official birth around the 1950 s, corresponding to the dawn of efforts to explore ways of attributing intelligence to machines (Sandewall, 2014). While some scholars place the first intelligent machine questions back in antiquity, with Aristotle and Sinclair (1962), proposing that if “every tool we had could perform its task, either at our bidding or itself perceiving the need, and […] play a lyre of their own accord, then master craftsmen would have no need of servants nor masters of slaves.” While others place such questions after the Renaissance period, with the advent of the scientific method (Bibel, 2014; Williams, 2002).

In fact, the roots of modern AI can be traced back to the legendary Greek scientists and their efforts to track and predict the lunar and solar eclipses, as well as solar, lunar and planetary positions. Known as the Antikythera mechanism, it is the ‘world’s first computer’ and is more than 2,000 years old. This astronomical calendar, or calculator, was discovered in a shipwreck off the coast of Crete in 1901 and predates other known examples of similar technology by more than 1,000 years (Dennehy, 2020).

Independent of when this quest may have begun, it is essential to note that these efforts have included the development of machines capable of demonstrating a variety of human behaviors and possessing human-like cognitive, emotional, and social intelligence (Haenlein & Kaplan, 2019). Consequently, there are debates about what AI actually means and entails.

While we agree that it is essential to differentiate between different kinds of AI and not simply assume that it ought to entail machine learning, which in itself is a multifaceted concept (Ågerfalk, 2020), a detailed elaboration on the specific origins and different angles around this debate is beyond the scope of this study. However, a brief exploration around treatments of AI reveals that the topic has been mainly considered in the context of machine learning applications. For example, as part of efforts to enhance: knowledge management systems (Irani et al., 2005; Kettinger & Li, 2010; Mentzas, 1991; Shah et al., 2007; Topi et al., 2006), information systems’ security/privacy (Brinton et al., 2016; Hsu, 2009; Lowry et al., 2017; Müller et al., 2016), decision support systems (Lederman & Johnston, 2011; Li et al., 2014; Lynch & Gregor, 2004), design science research (Kuechler & Vaishnavi, 2008) and natural language processing (Evangelopoulos et al., 2012). In light of this, and in order to bound this study’s scope, we are concerned with AI entailing the use of algorithms for advancing machine learning in the context of recommender systems (i.e., AI-based recommendations).

Understood this way, we agree with Fonseka (2017) that academics are in arrears of holding AI accountable. In particular, as Ågerfalk (2020, p. 5) suggests, “there are good reasons to worry about misuses of AI,” given its potential to perpetuate society’s inequalities and injustices through implicit biases due to race, gender, and sexual orientation (Manyika et al., 2019). Insofar as for instance, recently, researchers found that COMPAS, an AI-based recommender software used to assign recidivism scores (and help predict which convicted criminals were likely to re-offend), labeled Blacks who did not actually re-offend as a higher risk at nearly twice the rate as Whites. While Whites who went on to commit other crimes were much more likely to be labeled lower risk than Blacks. In essence, COMPAS produced double the number of false positives for Blacks than for Whites (Angwin et al., 2016). Another example is Amazon’s recruitment tool, which produced AI-based recommendations that significantly favored men over women for technical jobs (Dastin, 2018). This happened because the depth, range, and scope of the data used to train algorithms were critical for the accuracy of the subsequent classification and recommendation tasks provided by the AI tools. Because of this, the dataset of convicted criminals used to train COMPAS had more Blacks than Whites, while the training data used by Amazon’s recruitment tool was comprised of résumés mostly submitted by men (ibid.).

It seems troublesome that a society’s implicit biases (in perhaps too many realms of daily life) may be exacerbated through the use of AI-based recommendations (Howard & Borenstein, 2018). However, an often-overlooked aspect of AI-based recommendations pertains to the degree to which users -such as the United States’ courts (in the case of COMPAS) and/or human resource managers (in the case of Amazon)- are likely to believe in (or rather question) biased AI-based recommendations. Thus, a reasonable question to ask is whether some individuals would be more likely to question an AI-based recommendation if they happened to perceive it as biased (specifically in terms of race or gender).

To our best knowledge, IS scholars have not yet investigated this particular issue in relation to individuals’ cultural values. Even though, a recent study that focused on internet-mediated social networks established a link between espoused national cultural values and their perception of what ought to be appropriate and inappropriate social network behaviors (Gupta, 2021; Gupta et al., 2018). Furthermore, prior IS research also identified the role of espoused national cultural values in explaining various IS-oriented phenomena, such as individuals’ technology acceptance (Srite & Karahanna, 2006), internet shopping behaviors (Sia et al., 2009), continued intention to use mobile applications (Hoehle et al., 2015), internet security behaviors (Chen & Zahedi, 2016), and reactions in online reviews (Hong & Kim, 2016).

The central premise of all these studies is that individuals develop their cultural self by acquiring the perception of what is good or bad from an early age based on how others, such as parents and teachers, reward and punish their behaviors (Gelfand et al., 2011; Hofstede et al., 2010). As well as based on their experiences, from being present in society and watching how others behave around them. Along a similar vein, we aim at elucidating the effects of individuals’ cultural values on AI questionability due to perceived bias, which we understand and operationalize here as the extent to which individuals are likely to question racially or gender biased AI-based recommendations. Consequently, this study is guided by the following research question **(RQ)**:

- Do individual-level cultural values affect the extent to which individuals would question AI-based recommendations due to perceived racial or gender bias?

To answer this research question, we will follow Srite and Karahanna’s (2006) model of individual-level cultural values, derived from Hofstede’s (1982) cultural framework. At the individual-level, culture could be considered as a measure akin to an individual’s personality. Specifically, this study examines the effects of five cultural values (collectivism-individualism, power distance, masculinity-femininity, uncertainty avoidance, and long/short-term orientation) on the extent to which individuals are likely to question AI-based recommendations due to perceived racial or gender bias. This should matter to those who act (or are expected to act) based on the recommendations suggested by AI tools, and especially to the individuals who may be impacted by the decisions taken by organizations relying on AI-based recommendations (i.e., organizations utilizing AI-based recommendations), as well as to society as a whole. In addition, we engage in this endeavor since those whose identity (in terms of race or gender) could be threatened -by biased AI-based recommendations- may end up altering previously well-established product/brand preferences (White & Argo, 2009). Which could in turn instigate negative word-of-mouth and/or protests against firms (Romani et al., 2013) or organizations that may have inadvertently acted upon biased AI-based recommendations (even in cases when these recommendations could be shown to have come from third parties providing unintentionally ill-conceived AI-based recommender systems).

The remainder of this paper is structured as followed. First, theoretical background to AI-recommender systems is presented. Next, development of the hypothesis, followed by the methodology is provided. Then, the results are presented. Discussion, implications, and future research is discussed. The paper ends with a conclusion.

## Literature Review

In this section we will first discuss additional instances of biased AI-based recommendations along with their relation to causes of algorithmic bias. We will then delve into how AI-based recommendations, and ubiquitous computing in general, have been generating doubts and questions in many fields. And then finalize by providing detailed justification for the cultural construct employed here.

### AI-based Recommendations and Algorithmic Bias

In addition to the examples of biases discussed in the "Introduction" section, evidence of bias in AI-based recommendations has been undertaken in the context of: healthcare, in which misguided algorithmic predictions were generated as they were based on the level of healthcare expenditures instead of the risk of illness (Obermeyer & Mullainathan, 2019). Of online advertising of STEM (Science, Technology, Engineering, and Mathematics) jobs, which were displayed differently to men and women (Lambrecht & Tucker, 2019), and of financial services, in which AI-enabled credit scoring mechanisms led to higher interest rate loans for minorities (Fuster et al., 2018).

The above are not the only instances of AI-based recommendations exhibiting biases. Surely, other examples exist in many other domains, for example: screening of passengers at airports, online hotel/travel booking apps, blocking of content on social media networks, among others. Admittedly, individuals exposed to AI-based recommendations may (or may not) detect bias in them. However, for purposes of this study what matters most is that, if/when they do, efforts should be devoted to helping elucidate who would be more likely to question biased AI-based recommendations. Because of this, this study focuses on examining whether espoused national cultural values play a role in the extent to which individuals are likely to question AI-based recommendations due to perceived racial or gender bias.

From the information systems (IS) artifact design perspective, biased AI-based recommendations can emerge from algorithmic unfairness (Bellamy et al., 2018; Cowgill & Tucker, 2020; Pessach & Shmueli, 2020). Sources of algorithmic unfairness can be categorized as *bias in algorithmic predictions* (due to unrepresentative training samples, mislabeling of outcomes in training samples, coding/programming bias, and algorithmic feedback loops), and *biased algorithmic objectives* (related to decision thresholds that may limit/promote diversity, spillovers emerging from biased group-level outcomes, and a trade-off between the exploration of new information and exploitation of existing information) (Cowgill & Tucker, 2020). Farnadi et al., (2018) highlight that algorithmic bias may emerge from systematic bias present in data (owing to societal/historical features), as well as from feedback loops when biased recommendations get displayed by a recommender system and then get further entrenched, due to the fact that there is an “increase in probability for the item to be retained in the system” (p. 18). In the context of this study, we consider algorithmic bias in AI-based recommendation systems as furthering the marginalization of individuals because of their race or gender.

### Questioning AI-based Recommendations

Indeed, scholars have been posing critically important questions regarding ubiquitous computing since at least the last quarter century (Araya, 1995). In particular, in relation to losing a sense of otherness that tends to accompany the minimization of human involvement and interactions, which is in part what has occurred in the examples discussed in the introduction as well as in the previous section. This forewarning is becoming increasingly relevant and critical nowadays. For instance, in relation to AI-based recommendations and decisions made by automated vehicle technologies responding to unavoidable road traffic accidents (Cunneen et al., 2019). Concerns have emerged about the use of AI-based recommendations related to the accuracy of medical diagnosis and prognosis (Jain et al., 2020; Thrall et al., 2021), how inaccurate AI-based healthcare recommendations may adversely impact levels of trust between physicians and patients (Hoeren & Niehoff, 2018), as well as new technology acceptance levels among users (Fan et al., 2018). While technology adoption and acceptance issues are brought up as potential adverse consequences of questioning AI-based recommendations, but the discussion of how espoused national cultural values might affect levels of healthcare IS artifact adoption or acceptance is outside the scope of this study.

Questions regarding the use of AI-based recommendations have also emerged in automating law and policy procedures (Hartzog, 2017), and while simulating urban and regional land-use dynamics (Grinblat et al., 2016). As well as in the realms of art (Lyons, 2020), architecture (Kirsch, 2019; West & Burbano, 2020) and even while considering the visual and procedural aesthetics of computerized games (Rementeria-Sanz, 2020). In sum, academics from various disciplines have been sounding the alarm about the various ways in which things could have, and actually have, gone wrong. Which is why, once again, it becomes increasingly relevant to study behavioral factors affecting the extent which individuals are likely to question AI-based recommendations, in general, but especially (in our estimation) when they happen to be perceived as racially and/or gender biased. The role of culture, specifically individual level cultural values are discussed in the next section.

### National Culture and Espoused National Cultural Values

The role of culture, a complex concept, in IS studies has slowly received the attention of IS scholar, the research involving culture remains challenging. The main challenge pertains to the definition of culture. There are as many as 150 definitions of “culture” in the literature, yet there is no consensus on one (Kroeber & Kluckhohn, 1952). For example, Hill (2005) describes culture as a system of values and norms that are shared among a group of individuals and that when taken together constitute a design for living, while Hofstede (1980) calls culture “the collective programming of the mind which distinguishes the members of one human group from another” (p. 260).

Another challenge is the existence of various cultural frameworks (Gupta & Gupta, 2019), and consequently the presence of multiple measures of culture (e.g., Hall and Hall, 1976; Hofstede, 1980; House et al. 2002; Gelfand et al., 2011). Each available cultural framework provides a unique way to enhance our understanding of the multifaceted culture construct. Hofstede’s framework remains the most cited in IS literature as IS scholars have actively relied on this framework to investigate various technological phenomena (Chu et al., 2019). For instance, Tan et al. (1995) explored the relationship between cultural values and group support systems. McCoy et al. (2007) studied how technology adoption may vary across cultures. Hoehle et al. (2015) investigated the effects of cultural values on individuals’ continued intention to use mobile applications. George et al. (2018) examined the impact of the interaction between cultural values and different communication media on individuals’ ability to detect deception successfully. Moreover, a recent study has suggested that national cultural values may affect the extent to which countries utilize non-pharmaceutical technological interventions (NPTI) in mitigating the spread of coronavirus 2019 (COVID-19) pandemic (Gupta et al., 2021).

Consistent with this stream of cross-cultural IS research, in this study, we use Hofstede’s model as the basis to derive our study hypotheses. Specifically, Hofstede’s (2011) framework consists of five dimensions: individualism-collectivism, power distance, masculinity-femininity, uncertainty avoidance, and long/short-term orientation.

Hofstede’s model is the preferred choice of most cross-cultural researchers; however, when it comes to the operationalization of the cultural dimensions, there are two schools of thought. There is one group that treats all people in a country or society as homogenous (Guo et al., 2020). The researchers in this group use the national scores (0‒100) for all cultural dimensions proposed by Hofstede et al. (2010) to compare cultural differences between countries. However, the opponents of this approach argue that, since Hofstede proposed these national cultural scores by aggregating individual responses in each country, these scores lack sensitivity to variance in individual responses (Cole et al., 2011; Roussin et al., 2016). In sum, designating the same score to all individuals in a country is not conceptually and practically appropriate. Moreover, since individuals in the same country likely inherit national cultural values to differing degrees, Srite and Karahanna (2006) presented the framework of espoused (individual-level) cultural values. Espoused cultural values refer to the extent to which an individual embraces the cultural values of his or her country. The espoused values framework has roots in cultural psychology and physiological anthropology that suggest a relationship between the cultural traits of an individual and his or her personality. Moreover, when the dependent variable in the study is measured at the individual level, it is recommended to avoid ecological fallacy by using individual-level measures of culture. However, since in this study we are interested in the extent to which individuals would be likely to question the outcomes of AI-based recommendations when perceived as racially and gender biased -which again is how we understand and operationalize AI questionability here, and will use as dependent variable, and thus from now on we shall simply refer to it as such- is an individual-level concern. It is not advisable for us to employ a national level culture construct, insofar as it entails a macro-level phenomenon (Hoehle et al., 2015; McCoy et al., 2005; Srite & Karahanna, 2006; Straub et al., 2002). Consequently, in this study we will analyze all data at individual level. We shall now discuss each cultural dimension and how it may relate to AI questionability in an effort to justify our hypotheses.

## Hypotheses

### Collectivism-individualism

The collectivism-individualism dimension describes the extent to which individuals value group-orientation over self-orientation. Strong group-oriented behaviors reflect collectivism, while individualism (i.e., the opposite of collectivism) is manifested in behaviors where the self is more important than others. Stated simply, collectivism places emphasis on “we, us, and our,” whereas individualism values “I, me, and myself” (Agrawal & Maheswaran, 2005; Kumashiro, 1999). This perceived feeling of “we-ness” is what differentiates people with collectivistic traits from individualists.

Collectivistic cultural values are characterized by the presence of strong, cohesive in-groups, which consist of others perceived to be similar to oneself. Furthermore, collectivists have a strong sense of community, loyalty, respect, and trust towards the other members of their in-group. A family, village, nation, organization, religious group, soccer team, and student body are examples of in-groups (Triandis, 1996). By comparison, individualists are focused on doing their own things. They value autonomy and are not obligated to trust and respect others the same way as those with collectivistic cultural traits.

We suggest that if collectivists perceive AI-based recommendations to be biased, they are more likely to question it. Due to their focus on the shared success and welfare of the group, collectivists will likely evaluate AI-based recommendations concerning others in society rather than just linking the outcome to oneself. Their high sense of community and respect towards others make them more equipped to assess the wide-ranging implications of discriminatory AI-based recommendations, resulting in the questioning of any perceived unfairness in the outcome. Thus, we posit:

- H1: Increasing collectivism will lead to high AI questionability (increasing individualism will lead to low AI questionability)*.*

### Power Distance

The dimension of power distance deals with the extent to which individuals accept and expect that power is distributed unequally in society. While inequality, in general, represents societal divisions due to socioeconomic status (i.e., education, income, and occupation), the term “power,” in addition to an individual’s socioeconomic status, may signify someone’s influence due to his or her social and/or political affiliation, race, caste, age, prestige, or intellectual ability. Hofstede (1980) argues that stratifications exist in all societies; however, some are more unequal than others.

High power distance cultural values maintain that inequality exists, and they do not perceive it as a problem. Everyone has a place in society, and thus, it is acceptable for some to be privileged (and underprivileged) in society. High power distance values imply obeying those with power, for example, the elderly due to their age and one’s superiors due to their organizational titles. Arguing with superiors or presenting a differing opinion is not encouraged and is often looked down upon. A good manager is one that performs difficult tasks and delegates repetitive and mundane tasks to subordinates. Moreover, managers seeking feedback or advice from their subordinates are considered weak and ineffective. It is also acceptable for senior-level managers to earn a significantly higher income than lower-level employees. In sum, those with less power must show deference to those with more power in society.

By comparison, low power distance cultural values advocate reducing the perception of power by allowing everyone to be treated equally. It is not customary for individuals to agree with others just because they have more influence due to their socioeconomic status, higher-level position, or political ranks. Everyone is encouraged to share their perspectives freely, even if they contradict the views of those with more power. Consequently, with regards to questioning a biased AI-based recommendation, we believe power distance will have a negative effect on it, namely: individuals will be more likely to question biased AI-based recommendations with decreasing power distance. Conversely, individuals with high power distance cultural values will exhibit lower AI questionability due to bias insofar as the presence and acceptance of inequality are intrinsic to this cultural dimension. Therefore, we propose:

- H2: High power distance values will lead to low AI questionability (low power distance values will lead to greater AI questionability).

### Masculinity‒Femininity

Like collectivism-individualism, masculinity and femininity are the opposite ends of the same cultural spectrum. While masculinity captures the extent to which individuals in society value assertiveness, heroism, and achievement, femininity emphasizes nurturing, quality of life, and modesty. Masculine cultures tend to be highly performance-oriented, while feminine cultures value a good consensual working relationship with others. Hofstede (1980) argues that while the masculinity-femininity dimension may look similar to biological sex (male/female), there is an important difference between the two. For instance, the “sex” categorizes individuals either into male or female at the time of birth based on the presence (or absence) of different biological factors (e.g., Y chromosome, reproductive gland) (Knox & Schacht, 2012). On the other hand, the cultural dimension of masculinity-femininity represents social gender. Thus, a biological male may have feminine or masculine cultural values and vice versa.

Masculinity-oriented individuals are driven by competitiveness, where their success is evaluated on objective performance criteria. For example, prior IS research found that individuals with masculine cultural values assessed the effectiveness of new technology based on the degree to which it improved their job performance and facilitated “achievement of work goals and advancement” (Srite & Karahanna, 2006; p. 685). Given their focus on practicality, masculine cultures are called “tough,” while feminine cultures are referred to as “tender.”

We posit that masculine cultural values are more likely to question biased AI-based recommendations as these cultural values emphasize the need for meritocracy. Masculinity-oriented individuals believe that the credit should be given to the rightful person because one earns one’s success. The inherent explicitness in the masculine-oriented cultural values will likely dictate the individuals to question an AI-based recommendation if they perceive any discrimination against a deserving individual. Therefore, we suggest:

- H3: Increasing masculinity will lead to high AI questionability (increasing femininity will lead to low AI questionability).

### Uncertainty Avoidance

This dimension measures the extent to which individuals in a society are risk-averse versus risk-tolerant. Those with high uncertainty avoidance values have a propensity to feel threatened while dealing with unplanned events. They would want to minimize any degree of ambiguity in their lives and make the future as evident as possible. Therefore, high uncertainty avoidance cultural values endorse formal rules and regulations in organizations, institutions, and relationships to prevent uncertainty in everyday situations. By comparison, those with low uncertainty avoidance values have a high tolerance for risk and thus are not intimidated when presented with unexpected circumstances. It is not that individuals with high uncertainty avoidance values are terrified of taking a risk; however, when they do have to take a risk, they would instead opt for a risk that is known rather than unknown (Hofstede, 2003).

When individuals with high uncertainty avoidance cultures come across a biased AI-based recommendation, they will likely question it. This is because of the inherent unforeseen risks associated with believing in the AI-based recommendation that seems discriminatory. By comparison, the risks associated with the unknown do not affect the behaviors of those with low uncertainty avoidance cultures. The objective of putting together rules and structures in high uncertainty avoidance cultures is to enable smooth functioning of everyday activities in organizations and society. Individuals with high uncertainty avoidance values prefer clarity and have a low tolerance for irregular or deviant behaviors (Hofstede, 2011). These individuals may further feel anxious and stressed out when they do not obtain the outcome that they were expecting.

Consequently, the perceived bias in the AI-recommendation can be considered a significant deviance from the outcome that risk-averse individuals would expect, which likely makes them uncomfortable. There is also some evidence that individuals with high uncertainty avoidance cultural values tend to hesitate while using novel products and technologies (Hofstede et al., 2010). Thus, they may consider the mere act of using a novel technology risky, such as AI-based recommender systems, which could, in turn, exacerbate their questioning of AI-based recommendations that seem biased. Thus, we posit:

- H4: High uncertainty avoidance cultural values will lead to high AI questionability (low uncertainty avoidance cultural values will lead to low AI questionability).

### Long/Short-Term Orientation

This cultural dimension measures the extent to which individuals in a society depend on long-standing traditions and past historical events to make decisions about the present and future. Long/short-term orientation was not a part of the initial cross-cultural model suggested by Hofstede. It was added later as the fifth dimension to the model based on the work of Bond (1988). Due to its roots in Confucianism philosophy, initially, this dimension was not well received in the cross-cultural community (Fang, 2003). However, over time, long/short-term orientation has been established as an essential cultural dimension capable of explaining individuals’ behaviors (Hofstede et al., 2010). Moreover, several IS scholars (George et al., 2018; Hoehle et al., 2015) advocate for the use of this dimension in cultural studies.

Long-term oriented values are based on the premise that everything is temporary, and the change is inescapable. By comparison, due to their deep-rooted respect for past traditions, those with short-term oriented values are reluctant to change. Long-term oriented values are reflected in careful management of money, being persistent despite criticisms, and willingness to give up today’s fun and leisure for success in the future. In contrast, personal stability and expectation of quick results are important short-term oriented values. Given its forward-looking focus, long-term orientation is called a pragmatic cultural dimension, while short-term orientation is referred to as the normative dimension. Thus, it is not surprising that long-term orientated values are found to foster innovation (Van Everdingen & Waarts, 2003).

To believe in an AI-based recommendation, individuals would also need to believe in the innovative potential of AI in general. Given their openness to change and willingness to try out new technologies, long-term oriented individuals are likely to believe in the potential of AI-based technologies and their recommendations. Moreover, they will likely have a low AI questionability in general. Now, there is also a fundamental philosophical difference between long-term and short-term orientations concerning what is good and evil. Long-term orientation prescribes that the definition of good versus evil may change depending upon the circumstance, while the short-term oriented societies have universal guidelines for differentiating between the two. According to long-term orientation, “If A is true, its opposite B can also be true,” while short-term oriented cultures believe in “if A is true, its opposite B must be false” (Hofstede et al., 2010; p. 251). In sum, long-term oriented individuals are more likely to give AI-based recommendations the benefit of the doubt even if they seem biased. Meanwhile, in light of their clear distinction between what is good and evil, short-term-oriented individuals will likely exhibit high AI questionability due to perceived bias. Therefore, we propose:

- H5: Long-term oriented cultural values will lead to low AI questionability (short-term oriented cultural values will lead to high AI questionability).

In sum, we hypothesized that increasing collectivism, masculinity, and uncertainty avoidance will lead to high AI questionability, while power distance and long-term oriented cultural values will result in low AI questionability.

## Research Methodology

### Participants and Data Collection

Data were collected using Amazon Mechanical Turk (MTurk), a crowdsourcing marketplace where individuals or businesses (referred to as requesters) post jobs to registered MTurk workers who volunteer to complete the published jobs and earn monetary incentives in return. The jobs include anything from conducting simple data validation and research to more subjective tasks like survey participation, content moderation, and more (MTurk, 2020). Prior research indicates that data collected from MTurk are of high quality and capable of producing breakthroughs in research (Lowry et al., 2016). More recently, IS scholars have relied on MTurk to test their study hypotheses (Maier et al., 2019; Marett et al., 2017).

### Measurement

#### AI Questionability Due to Perceived Bias

Seven different scenarios (see Table 1) to capture AI questionability were created and reviewed by AI experts. Each of the seven scenarios reflected a clear real-world example of a biased AI-based recommendation. Participants first read the following two scripts, which was modified to imply racial bias (after controlling for gender) and gender bias (after controlling for race).

- *Script 1: AI questionability due to* *racial* *bias -* Let us imagine that you and a friend have the same gender, age, as well as practically identical educational and professional achievements but have a different race. How likely are you to question the following outcomes?
- *Script 2: AI questionability due to* *gender* *bias -* Let us imagine that you and a friend have the same race, age, as well as practically identical educational and professional achievements but have a different gender. How likely are you to question the following outcomes?

Participants then chose an option on a 5-point Likert-type scale where 1 meant Highly Unlikely, and 5 meant Highly Likely. All scenarios were randomized such that the order of the seven scenarios was different for every participant.

**Table 1:** Scenarios depicting AI biases due to gender and race

#	How likely are you to question the following outcomes? (1 = Highly Unlikely; 5 = Highly Likely)	AI scenario (supporting references from news media/published research)
1	You are both applying for the same financial product (such as a credit card or home mortgage/loan) on the same bank app/website using your own devices. You notice the products that are offered to your friend charge higher interest rates than those offered to you.	Bias in financial services (Hamilton, 2019) (Fuster et al., 2018)
2	You are both looking for similar jobs on the same employment app/website using your own devices. You notice the jobs that are offered to your friend usually have lower-paying salaries than those offered to you.	Bias in recruitment (Dastin, 2018) (Lambrecht & Tucker, 2019)
3	You both have the same nationality and are at the airport going through the same automated immigration kiosk that uses face recognition technology to verify travelers’ identity. The automated immigration kiosk directs your friend to see an immigration officer while you are cleared to go through.	Bias in facial recognition software used to screen travelers (Tate, 2019)
4	You are both booking a similar hotel room using the same hotel booking app/website using your own devices. Hotel rooms offered to your friend have higher prices than those offered to you.	Bias in online hotel bookings (Hannak et al., 2014)
5	You are both booking the same flight using the same travel booking app/website on your own devices. Flights offered to your friend are costlier than those offered to you.	Bias in online flight bookings (Hannak et al., 2014)
6	You both regularly write posts on similar topics on the same social network service (for instance, Facebook). Your friend’s posts are found objectionable (that is, flagged for removal) more often by the social network service than those posted by you.	Bias in blocking online content (Ghaffary, 2019)
7	You both have similar diets, daily routines, and are feeling just fine. You are both using an automated health assessment app on your own devices that involves interacting and answering questions using voice recognition. The automated health assessment app suggests that your friend is at a higher risk of contracting the flu, but not you.	Bias in healthcare space (Gershgorn, 2018) (Obermeyer & Mullainathan, 2019)

We first conducted a pilot survey of 60 MTurk users to ensure the readability and clarity of the seven scenarios pertaining to racial and gender bias. Following this, the main study was administered, and 387 completed responses were collected using MTurk in the United States. We ensured that those who participated in the pilot survey were excluded from participating in the main study. Of the 387 users, 237 were males, and 150 were female participants. The average age of a participant was 38.26 years, and each participant received $1.50 for his or her participation.

#### Cultural Values

**Fig. 1:**
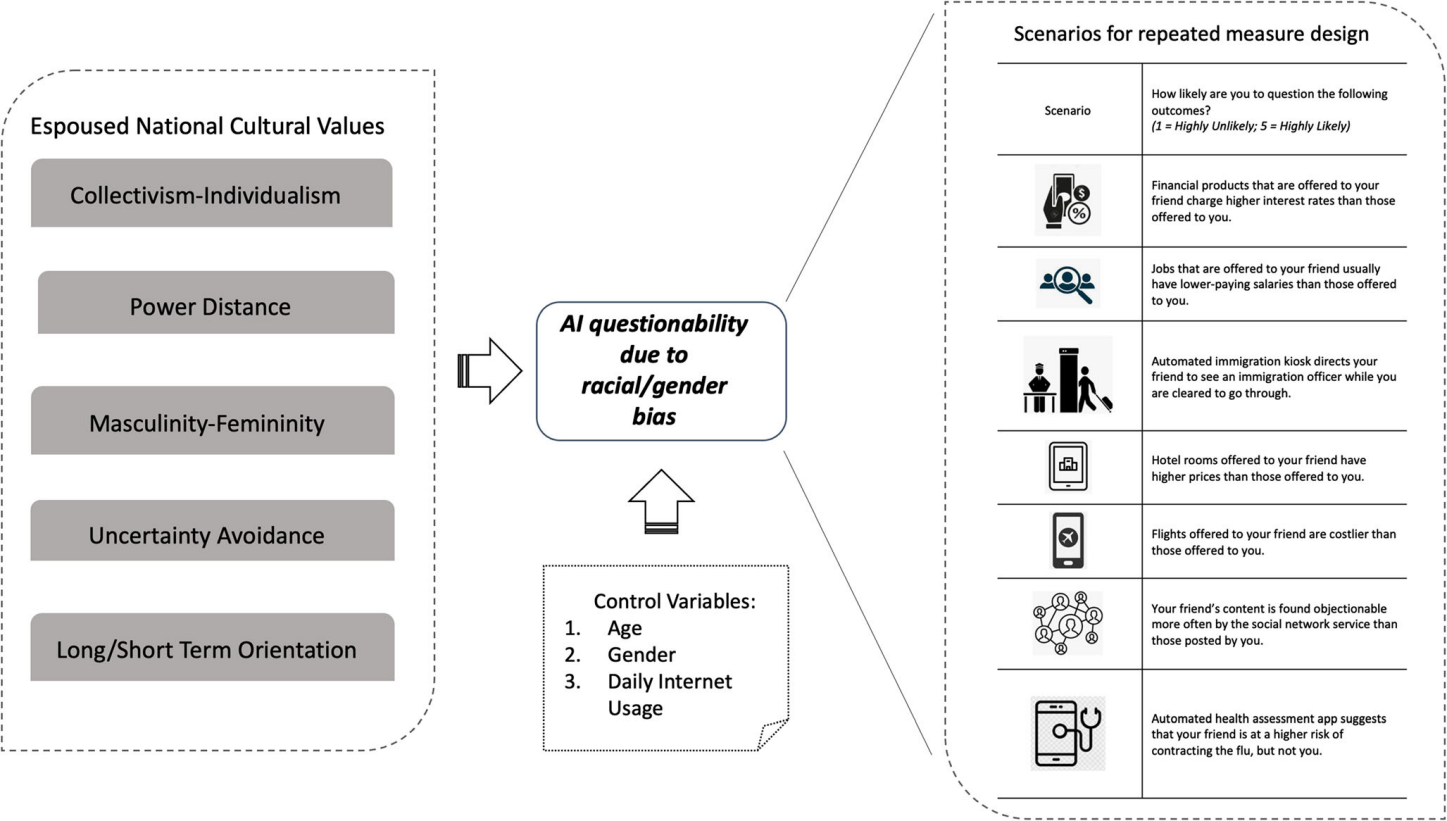
Scenario-based research model

To capture cultural variables, we employed established measures available in the literature. The dimensions of collectivism-individualism, power distance, masculinity-femininity, and uncertainty avoidance were assessed using previously validated scales presented by Srite and Karahanna (2006). To measure long-term orientation, we utilized the scale suggested by Yoo et al. (2011). We also measured participants’ age, gender, and daily internet usage as control variables. Figure 1 below details the research methodology followed, in particular how outcomes of biased AI-based recommendations were used to explore the influence exerted by individual level cultural values, after controlling for type of bias, age, gender and internet usage on AI questionability. The seven scenarios with situational outcomes are used to explore the influence exerted by espoused national cultural values (after controlling for age, gender and internet usage) on AI questionability due to perceived racial/gender bias.

## Analysis and Key Findings

We first conducted a confirmatory factor analysis of the measures of the five cultural constructs in AMOS using IBM SPSS (Version 21). After removing the measures with low loadings, the resultant model showed a good fit according to the recommended cutoff values: χ2 = 250.92, df = 125, χ2/df = 2.00 (between 1 and 3), RMSEA = 0.05 (below 0.08), SRMR = 0.05 (below 0.08), and CFI = 0.97 (above > 0.90) (Hair et al., 2006; Kline, 2015). All factor loadings are shown in Table 2.

**Table 2:** Factor loadings

PD_1	.86	0.35	0.52	− 0.10	0.00
PD_2	.85	0.40	0.62	− 0.14	0.10
PD_3	.85	0.41	0.57	− 0.07	0.06
PD_4	.82	0.35	0.50	− 0.09	0.13
PD_5	.78	0.32	0.51	0.02	0.12
PD_6	.74	0.19	0.46	0.12	0.16
COL_1	0.38	.90	0.44	− 0.08	0.16
COL_3	0.31	.90	0.41	− 0.11	0.09
COL_2	0.42	.86	0.46	− 0.06	0.24
MAS_2	0.62	0.46	.93	− 0.13	0.22
MAS_1	0.62	0.45	.92	− 0.09	0.16
MAS_3	0.64	0.47	.92	− 0.11	0.21
UA_1	− 0.05	− 0.02	− 0.04	.83	0.22
UA_3	− 0.06	− 0.11	− 0.14	.80	0.32
UA_2	0.04	− 0.08	− 0.06	.77	0.33
LTO_3	0.06	0.09	0.08	0.41	.83
LTO_1	− 0.03	0.13	0.16	0.32	.80
LTO_2	0.23	0.20	0.26	0.14	.80

Reliabilities were assessed using Cronbach’s α values (see Table 3), which were above the recommended value of 0.70 for all constructs (Hair et al., 2006). We further calculated variance inflation factors (VIF) to assess multicollinearity. All VIF values were below 3.3, indicating multicollinearity was not a concern in this study (Petter et al., 2007).

**Table 3:** Measures and descriptives

Construct		Item	Mean	SD
Collectivism (α = 0.87) (Srite & Karahanna, 2006)	COL1	Group success is more important than individual success.	2.99	1.25
	COL2	Being loyal to a group is more important than individual gain.	3.06	1.24
	COL3	Individual rewards are not as important as group welfare.	3.07	1.30
	COL4	Being accepted as a member of a group is more important than having autonomy and independence. ^1^	NA	NA
	COL5	Being accepted as a member of a group is more important than being independent. ^1^	NA	NA
Power Distance (α = 0.90) (Srite & Karahanna, 2006)	PD1	Managers should make most decisions without consulting subordinates.	2.83	1.29
	PD2	Managers should be careful not to ask the opinions of subordinates too frequently; otherwise, the manager might appear to be weak and incompetent.	2.74	1.32
	PD3	Decision-making power should stay with top management in the organization and not be delegated to lower-level employees.	2.81	1.28
	PD4	Employees should not question their manager’s decisions.	2.70	1.32
	PD5	A manager should perform work that is difficult and important, and delegate tasks that are repetitive and mundane to subordinates.	3.01	1.24
	PD6	Higher-level managers should receive more benefits and privileges than lower-level managers and professional staff.	3.26	1.28
Uncertainty Avoidance (α = 0.73) (Srite & Karahanna, 2006)	UA1	Rules and regulations are important because they inform workers what the organization expects of them.	4.20	0.75
	UA2	Order and structure are very important in a work environment.	4.22	0.90
	UA3	It is important to have job requirements and instructions spelled out in detail so that people always know what they are expected to do.	4.18	0.86
	UA4	It is better to have a bad situation that you know about than to have an uncertain situation which might be better.^1^	NA	NA
	UA5	Providing opportunities to be innovative is more important than requiring standardized work procedures.^1^	NA	NA
LTO (α = 0.80) (Yoo et al., 2011)	LTO1	Long-term planning	4.04	0.92
	LTO2	Giving up today’s fun for success in the future	3.66	1.01
	LTO3	Working hard for success in the future	4.06	0.88
	LTO4	Careful management of money (Thrift) ^1^	NA	NA
	LTO5	Going on resolutely despite opposition (Persistence)^1^	NA	NA
	LTO6	Personal steadiness and stability^1^	NA	NA
MAS (α = 0.91) (Srite & Karahanna, 2006)	MAS1	It is preferable to have a man in a high-level position rather than a woman.	2.66	1.37
	MAS2	It is more important for men to have a professional career than it is for women to have a professional career.	2.48	1.44
	MAS3	Solving organizational problems requires the active forcible approach, which is typical of men.	2.61	1.41
	MAS4	There are some jobs in which a man can always do better than a woman.^1^	NA	NA
	MAS5	Women do not value recognition and promotion in their work as much as men do. ^1^	NA	NA

^1^ Item dropped from analysis due to low factor loadings and/or low reliability; M = Mean; α = Cronbach’s alpha; NA = not applicable as the item was dropped

As mentioned previously, each participant answered seven questions, each focusing on a distinct example of a biased AI-based recommendation. We conducted two repeated measure linear mixed model (LMM) analyses in SPSS (Version 21), first was for AI questionability (due to perceived racial bias) and the second with AI questionability (due to perceived gender bias). The LMM regression analysis is recommended where the perception of the participant (i.e., the dependent variable) is measured repeatedly by using multiple scenarios (Gupta et al., 2018). The LMM design requires the data to be set up in a long format such that there were seven rows of data per participant. Each AI scenario acted as a repeated measure in our analysis. The results of the two LMM regressions are shown in Table 4

**Table 4:** Regression results

Dependent Variable								
Source	AI Questionability (Racial Bias)				AI Questionability (Gender Bias)			
	Numerator df	Denominator df	F	Sig.	Numerator df	Denominator df	F	Sig.
Intercept	1	2682.04	14.25	P < .001	1	2686.61	14.80	P < .001
COL	1	2682.04	97.48	P < .001	1	2686.61	59.34	P < .001
PD	1	2682.04	0.10	ns	1	2686.61	0.68	ns
MAS	1	2682.04	35.42	P < .001	1	2686.61	83.02	P < .001
UA	1	2682.04	27.15	P < .001	1	2686.61	12.28	P < .001
LTO	1	2682.04	1.18	ns	1	2686.61	0.15	ns
Gender	1	2682.04	27.23	P < .001	1	2686.61	11.41	P < .01
Age	1	2682.04	8.37	P < .01	1	2686.61	1.60	ns
Internet Usage	1	2682.04	30.10	P < .001	1	2686.61	20.37	P < .001

Notes: ns = not significant; COL = Collectivism, PD = Power Distance, MAS = Masculinism, UA = Uncertainty Avoidance, and LTO = Long-term Orientation

### Racial Bias

For AI questionability (due to racial bias) as the dependent variable, we found support for three of the five hypotheses. Consistent with our stated hypotheses, increasing collectivism (β = 0.23, p < .001), masculinity (β = 0.16, p < .001), and uncertainty avoidance (β = 0.19, p < .001) led to an increase in participants’ questioning of the racially biased AI-based recommendations. Power distance and long-term oriented values had insignificant effects on AI questionability (race). All control variables were found significant. For gender (β = -0.26, p < .001), the pairwise comparisons indicated that female participants (Mean = 3.71, SE = 0.04) had higher mean AI questionability (race) than that of males (Mean = 3.45, SE = 0.03). AI questionability also increased with increasing participants’ age (β = 0.01, p < .01) and daily internet usage (β = 0.20, p < .001).

### Gender Bias

For AI questionability due to gender bias as the dependent variable, the results concerning cultural variables were similar as the earlier analysis. Three of the five hypotheses were supported. While increasing collectivism (β = 0.18, p < .001), masculinity (β = 0.24, p < .001) and uncertainty avoidance (β = 0.13, p < .01) led to an increase in participants’ questioning of the AI-based recommendations when they perceived the recommendation had a gender bias. The hypotheses regarding power distance and long-term orientation were found not significant. All control variables were significant, except for the age variable. For gender (β = -0.17, p < .01), the pairwise comparisons indicated that female participants (Mean = 3.55, SE = 0.04) had higher mean AI questionability (gender) than that of males (Mean = 3.38, SE = 0.03). As participants’ daily internet usage (β = 0.17, p < .001) increased, AI questionability (gender) also increased. A summary of the hypotheses results is provided in Table 5.

**Table 5:** Hypotheses results

Hypothesis	AI questionability (*Racial* bias)	AI questionability (*Gender* bias)	
**H1**: Increasing *collectivism* will lead to high AI questionability	Yes	Yes	
**H2**: High *power distance* values will lead to low AI questionability	*No*	*No*	
**H3**: Increasing *masculinity* will lead to high AI questionability	Yes	Yes	
**H4**: High *uncertainty avoidance* cultural values will lead to high AI questionability	Yes	Yes	
**H5**: *Long-term oriented* cultural values will lead to low AI questionability	*No*		*No*

Interestingly, the dimensions of power distance and long-term orientation had non-significant effects on AI questionability. A possible explanation is that the effects of individual-level power distance and long-term oriented cultural values may have been masked by the presence of other cultural values (i.e., collectivism, masculinity, and uncertainty avoidance). For the same reason, some studies examine the effect of one cultural value at a time on the dependent variable (e.g., George et al., 2018). To confirm our suspicion, we conducted additional analyses to assess the separate (individual) effects of power distance and long-term orientation on AI questionability. Both power distance and long-term orientated cultural values, in the absence of other cultural variables, had significant effects on AI questionability. Given that an individual’s cultural self depends to differing degrees on each of the five cultural dimensions, it is important to consider all cultural values in the analysis instead of focusing on one at a time.

There are also some interesting findings about the role of control variables. Regardless of the type of the bias, participants’ gender and their daily internet usage had significant effects on AI questionability. Particularly, females exhibit higher AI questionability due to perceived bias than males. It is understandable as the popular press is rife with articles of artificial intelligence being biased against women, thereby making females, in general, more suspicious of AI-based recommendations (Niethammer, 2020). Participants’ daily internet usage also led to an increase in AI questionability. Individuals consume AI-based recommendations on a daily basis while using the Internet-enabled applications. For example, recommendations about routes from map applications, movies and songs from online streaming companies, and products from e-commerce applications. In most of these cases, individuals know whether the recommendations are appropriate, and as found in our study, the ones who use the Internet actively are more likely question an AI-based recommendation when perceived as biased.

With respect to the age variable, we would think that with increasing age, individuals are likely to question AI-based recommendations, and more so when they are biased. This is because artificial intelligence may discriminate against individuals because of their (older) age. Some have cautioned that AI-driven tools may further enhance the problem of “ageism” in hiring where younger candidates are preferred to older ones (Kolakowski, 2019). Participants’ increasing age showed high AI questionability due to perceived racial bias; however, their age was not a significant factor in the questioning of gender-biased AI-based recommendations. We speculate that this might be the case as older adults could be less at odds with traditional gender roles and expectations.

## Discussion, Limitations and Future Research

Indeed, AI-based recommendations may discriminate against some members of society more than others, and this we contend ought to be one of the most worrisome aspects of ubiquitous computing and generalized automation. Even though scholars have also been concerned, albeit recently, with proposing governance mechanisms to prevent AI-related misuses and abuses (Floridi & Cowls, 2019; Zuiderveen Borgesius, 2020), there still are reasons for concern. One such concern that we examine in this study is the extent to which individuals, owing to their individual-level cultural values, would be likely to question AI-based recommendations when perceived as racially or gender biased. The findings suggest that cultural values affect AI questionability due to perceived bias. As such, this study’s findings offer several theoretical and practical implications.

### Theoretical Implications

In the past few years, while the technical aspects of AI and associated capabilities have garnered substantial interest from academics and practitioners (Mikalef & Gupta, 2021), the research focusing on individual behaviors in response to AI-based outcomes remains scarce (Nishant et al., 2020). Shrestha and Yang (2019) call the studying of bias and fairness in AI-based recommender systems as an important and emerging research area that merits special attention. By specifically investigating the relationship between culturally influenced individual behaviors and AI questionability, this research contributes to the nascent field of IS focusing on the people side of AI. Moreover, with increasing globalization and the global movement of people, the IS research advancing the role of cultural factors has become increasingly relevant in recent years (Warkentin et al., 2015). This study advances the case for more research grounded in cultural theory to explain IS-driven phenomena.

IS scholars tend to generally focus on a select few cultural dimensions while ignoring other important dimensions (Chu et al., 2019). As each cultural dimension captures a unique individual-level cultural characteristic, the findings of this study emphasize the need to consider different dimensions of culture while examining individuals’ behaviors towards novel and complex IS phenomena such as AI-based recommender systems. While some cultural values may be more salient than others; however, we can only explore this when all cultural values are considered in the analysis.

The study further makes a methodological contribution to the IS literature. While there is much debate around the unit of analysis of the culture construct, we followed the recommendations of Srite and Karahanna (2006) and recent studies (e.g., George et al., 2018; Guo et al., 2020; Gupta et al., 2019), and conducted the analysis with an individual being the unit. Examination of the standard deviations (SD) of the cultural variables with respect to their means, for example, masculinity (Mean = 2.58, SD = 1.31) and collectivism (Mean = 3.04, SD = 1.12), yields evidence of significant cultural heterogeneity among individuals, especially for a country as diverse as the United States; thereby, providing further support for employing cultural values at the induvial level (Hoehle et al., 2015).

### Practical Implications

The findings from our study offer practical insights for managers utilizing AI-based recommendations, individuals impacted due to biased AI-based recommendations, and organizations developing and utilizing AI-based recommendation systems for decision making.

#### Managers Utilizing AI-based Recommendations for Decision Making

With the advent of emerging technologies, such as AI, big data, and analytics, there has been a significant push towards incorporating data/analytics/AI-driven insights into managerial decision-making processes (Popovič et al., 2018). As Brynjolfsson and Mcafee (2017, p. 20) suggest, “over the next decade, AI won’t replace managers, but managers who use AI will replace those who don’t.” However, most AI-based technologies in their current form are not yet ready to replace human intelligence (Lee, 2018; O’neil, 2016), and thus there is an urgent need to find the right balance between managers’ reliance on AI-based recommendations and using their own assessment to make a fair, unbiased decision.

Our findings illustrate how a manager’s cultural self may affect the questionability (or believability) of biased AI-based recommendations. For example, imagine a manager with individualistic, low masculinity, and weak uncertainty avoidance cultural values distance values is utilizing an AI-tool, which may produce biased recommendations due to unrepresentative data used for training it). Based on the study’s findings, the likelihood of a discriminatory outcome in realm of this manager’s business would be exacerbated by the use of AI. Conversely, a manager with collectivistic, high masculinity, and strong uncertainty avoidance cultural values manager would likely question the validity of biased AI-based recommendations. Perhaps it is in light of this that managers may decide to avoid using AI tools and go back to a manual review of recommendations by humans (Cowgill, Dell’Acqua, & Matz, 2020).

#### Individuals Impacted Due to Biased AI-based Recommendations

We believe it is imperative to consider the perspective of those who may be discriminated against by decisions made leveraging biased AI-based recommendations. Recruitment is one business function where organizations have been increasingly employing AI-based tools to screen candidates (Bogle & Sankaranarayanan, 2012). Recent research suggests that job applicants feel anxious while applying for jobs with organizations that openly use AI in the hiring process (Van Esch et al., 2019). The applicants’ levels of anxiety can be further aggravated due to their cultural values and if they perceive that the AI-system employed to determine the qualified candidate is biased against them either due to their race or gender. Those affected may respond negatively towards corporate infractions by instigating negative word-of-mouth and protesting toward the corporation (Romani et al., 2013). In particular, individuals seem to be less forgiving when actions are personally relevant to them, which again ought to be the case when race and gender are involved (Trump, 2014).

#### Organizations Developing and Utilizing AI-based Recommendation Systems for Decision Making

Undoubtedly, it has become essential for today’s organizations to use AI; however, they also have a responsibility in identifying and implementing remedial actions to strengthen fairness and accountability in AI-based tools. For example, on the development side, IS researchers have been proposing ways of addressing bias through: special treatments for protected groups by modifying algorithmic learning objectives (Yao & Huang, 2017) or measuring weight deviations between protected and unprotected groups (Ning & Karypis, 2011). Also by ensuring diversity in recommended items that balance individual input preferences, utility of objects selected, as well as object ratios from various groups (Steck, 2018) or simply by equating the ratio of objects from different groups to input preferences (Tsintzou et al., 2018).

*Other* efforts have focused on promoting fairness beyond end-user protection (Abdollahpouri et al., 2019) by striving to include all parties involved in recommender systems (i.e., producer or AI package provider, intermediator or firm adopting AI package to provide recommendations, and consumers) or multisided fairness (Burke, 2017). This focus on multiple stakeholder fairness is of course reminiscent of Freeman (2010) and his strategic management propositions associated to stakeholder engagement, which in turn aligns with ethics (Weiss, 1994) as well as with Corporate Social Responsibility (CSR) (Elms et al., 2011). In light of the fact that biases can become culturally entrenched through simple information transmission (Hunzaker, 2016), and that IS (in particular recommender systems) may amplify and exacerbate this entrenchment, it seems encouraging that businesses could eventually consider measuring, reporting and advancing algorithmic fairness as part of their code of conduct and CSR efforts.

On the other hand, managers utilizing AI-based recommendations for decision making- might consider increasing awareness among employees about the possibility of inherent biases in their AI-based recommender tools, and institute processes to report unfair or biased AI-based recommendations. It may sound contradictory to encourage managers to adopt a data-driven decision-making process, while at the same time informing their employees of possible AI tool limitations. How to balance out these two opposing views is a challenging task, yet it is something that organizations willing to exploit the power of AI-based recommendations ought to consider. Google, for example, has just announced that its AI tool would no longer display gender-specific labels, such as man and woman, to thwart bias (Lyons, 2020). Indeed, a lot more needs to be done to improve the fairness in AI-based recommendations, yet it is promising that large firms, such as Google, are taking small steps that will bolster the adoption of AI-based tools in the future.

## Limitations and Future Research

Like any other research, this study has limitations, which also offer directions for future research. First, we specifically focused on racial and gender bias, even though there have been instances where AI discriminated against individuals based on their economic status, sexual orientation, age, physical appearance (weight), disability, and ideologies. Future research could extend this research by considering biases beyond race and gender. Second, we relied on Hofstede’s cultural framework, when applied at the individual level, to understand how individuals’ behaviors may affect the questionability of biased AI-based recommendations. Though this framework remains the most popular among cultural scholars in the IS field, future research may also test the proposed relationships in this study through the lens of other cultural frameworks (e.g., Schwartz, 1999; Hall & Hall, 1976; House et al., 2004). Third, recently a new dimension of indulgence versus restraint has been proposed in the cross-cultural literature. However, there is limited evidence about its role at the individual level in the business discipline, including IS. Moreover, this dimension is considered a measure of happiness in a society and does not directly relate to the context of the current study. Focusing on the five established cultural dimensions allowed us to reduce the questionnaire length and minimize participant fatigue. Future research could explore the relationship between indulgence versus restraint dimension and AI questionability, if they identify a theoretical justification. Fourth, all participants in this study came from the United States. As discussed earlier, there is significant variance in espoused national cultural values in the sample, yet in order to broaden the generalizability of the study findings, future research may consider including participants from other countries.

## Conclusions

As presciently stated by Araya (1995, p. 237): “as the power of technologies grows, it will become increasingly necessary to probe into the assumptions being made during their inception and into the possible consequences of their widespread application.” Algorithmic bias research and solutions point to the first part of the task (i.e., assumptions made at inception). Meanwhile, our research -aimed understanding how behavioral factors (such as cultural values) affect the extent to which individuals may question racially or gender biased AI-based recommendations- entails a step in finding ways to help mitigate the consequences of widespread application by leveraging human involvement and dispositions. In particular, our results evidenced a relationship between three individual-level cultural values (collectivism, masculinity, and uncertainty avoidance) and AI questionability due to racial or gender bias. These results can have implications for strengthening fairness in AI-based recommender systems, use of which has become ubiquitous in nearly all business functions. But research on the people side of AI, and on how humans could help exacerbate and/or mitigate unwanted consequences of AI in general has only just started.
